# Studies on the Synthesis of DMAP Derivatives by Diastereoselective Ugi Reactions

**DOI:** 10.3390/molecules16108815

**Published:** 2011-10-20

**Authors:** Hiroki Mandai, Shunsuke Irie, Koichi Mitsudo, Seiji Suga

**Affiliations:** Division of Chemistry and Biochemistry, Graduate School of Natural Science and Technology, Okayama University, 3-1-1 Tsushima-naka, Kita-ku, Okayama 700-8530, Japan

**Keywords:** multicomponent reaction, Ugi reaction, chiral DMAP, kinetic resolution

## Abstract

Diastereoselective Ugi reactions of DMAP-based aldehydes with α-amino acids and *tert*-butyl isocyanide were examined. The reactions of 4-(dimethylamino)-2-pyridine-carboxaldehyde with various α-amino acids afforded 2-substituted DMAP derivatives with low diastereoselectivity. On the contrary, reactions with 4-(dimethylamino)-3-pyridine-carboxaldehyde delivered 3-substituted DMAP derivatives with moderate to high diastereoselectivity. The combination of α-amino acid and DMAP-based aldehyde is thus important to achieve high diastereoselectivity. Kinetic resolution of a secondary alcohol using a chiral DMAP derivative obtained through these reactions was also examined.

## 1. Introduction

Multicomponent reactions are highly efficient in atom economical transformations in synthetic organic chemistry [1]. They can be used for constructing various libraries of compounds in medicinal chemistry. Among the multicomponent reactions, Ugi reaction, known as a four-component reaction, combines a carbonyl compound, an amine, a carboxylic acid, and an isonitrile to afford highly functionalized molecules [2,3]. In general, no activator is required and one-pot synthesis is possible. Asymmetric variants of the Ugi reaction [4] have been reported using chiral α-methylbenzylamines [5,6,7,8], ferrocenylamines [9,10,11,12], glycosylamines [13,14], α-amino acids [15,16,17,18,19,20,21,22] and β-amino acids [23] to deliver the Ugi adducts with good to high diastereoselectivity. However, the use of a chiral isocyanide [24,25] or carboxylic acid [26,27,28] as the chiral inducer usually confers no asymmetric induction in the Ugi reaction.

In the course of our research, we have been interested in utilizing an asymmetric Ugi reaction for the synthesis of chiral nucleophilic organocatalysts [29]. Among these catalysts, the chiral molecule 4-(dimethylamino)pyridine (DMAP) [30] is known as a versatile catalyst for various asymmetric transformations, such as kinetic resolution of racemic alcohols [31], desymmetrization of anhydrides [32], and inter or intramolecular reactions of oxazolones [33]. However, incorporating a chiral environment in the DMAP structure is still a challenging issue because of long synthetic steps required to obtain optically pure catalysts. We anticipated that by developing an efficient protocol for the diastereoselective Ugi reactions of DMAP-based aldehydes, one-pot synthesis of diverse chiral DMAP structures may be easily carried out simply by changing substrate combinations. Furthermore, highly functionalized and easily tunable chiral DMAP derivatives are attractive as potential highly active, enantioselective catalysts for asymmetric transformation. In this paper, we describe the synthetic studies of diastereoselective Ugi reactions using DMAP-based aldehydes and the use of the Ugi products as chiral nucleophilic catalysts.

## 2. Results and Discussion

### 2.1. Diastereoselective Ugi Reaction of 4-(Dimethylamino)-2-pyridinecarboxaldehyde *(**1**)*

We carried out the Ugi reaction of 4-(dimethylamino)-2-pyridinecarboxaldehyde (**1**), L-valine, and *tert*-butyl isocyanide in MeOH as the solvent and an external nucleophile (U-5C-4CR). We speculated that the formation of the new stereogenic center could be controlled by L-valine to afford the product as a single diastereomer through simple purification; the product could then be utilized directly as a chiral nucleophilic catalyst (chiral DMAP). Because the Ugi reactions of DMAP-based aldehydes were not reported, we proceeded with the optimization of the Ugi reaction conditions.

Because the Ugi reaction is a condensation reaction between organic components, the concentration of substrates may be important for obtaining the desired product in reasonable yield. Thus, we examined different substrate concentrations for the Ugi reaction (Table 1). The reaction of DMAP-based aldehyde **1**, L-valine, and *tert*-butyl isocyanide in MeOH was carried out at room temperature for 15 h. Lower concentrations (0.1 and 0.2 M) of **1** delivered the desired product **2a** in which MeOH was incorporated, even though almost no diastereoselectivity was observed (79% and 84% yields; entries 1 and 2, respectively). At 0.5 M concentration, the reaction was accelerated sufficiently to afford **2a** in 98% isolated yield with a 63:37 diastereomeric ratio (d.r.) (entry 3). Higher concentration (1.0 M) afforded a yield slightly inferior to that achieved by 0.5 M concentration (91%, 60:40 d.r.). On the basis of these results, 0.5 M substrate concentration was determined to be optimal for the model reaction.

To improve the diastereoselectivity of the Ugi product, we carried out reactions with various *α*-amino acids under the aforementioned conditions. The structure of the *α*-amino acid side chain might be important to control the newly formed stereogenic center.

**Table 1:** Ugi reaction of **1** using various substrate concentrations. 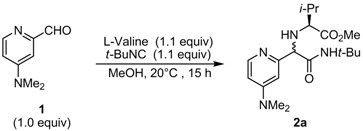

Entry	Concentration (M)	Yield (%) *^a^*	D.r. *^b^*
1	0.1	79	55:45
2	0.2	84	62:38
3	0.5	98	63:37
4	1.0	91	60:40

^a^ Yield of the mixture of diastereomers after column chromatography; ^b^ Diastereomeric ratio was determined by ^1^H-NMR analysis of unpurified products.

As shown in Table 2, we tested various commercially available *α*-amino acids. Reaction with L-t-leucine gave the desired product **2b** in 70% isolated yield with a 62:38 d.r., thus suggesting that the sterically congested side chain of the *α*-amino acid did not improve diastereoselectivity (entry 2 vs. 1). Other chiral sources, L-isoleucine, L-phenylalanine, and L-phenylglycine also showed similar diastereoselectivities (54%, 57%, 59% yield; 60:40, 55:45, 55:45 d.r.; entries 3*–*5, respectively).

**Table 2:** Ugi reaction of **1** with various *α*-amino acids. 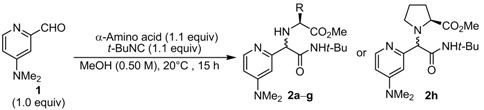

Entry	α-Amino acid	Product	Yield (%) *^a^*	D.r. *^b^*
1	L-Valine	**2a**	98	63:37
2	L- *t*-Leucine	**2b**	70	62:38
3	L-Isoleucine	**2c**	54	60:40
4	L-Phenylalanine	**2d**	57	55:45
5	L-Phenylglycine	**2e**	59	55:45
6	L-Threonine	**2f**	77	50:50
7	L-Serine	**2g**	85	53:47
8	L-Proline	**2h**	19	51:49

^a^ Yield of the mixture of diastereomers after column chromatography; ^b^ Diastereomeric ratio was determined by ^1^H-NMR analysis of unpurified products.

Furthermore, the reactions with L-threonine and L-serine, both having a hydroxyl group in the side chain, delivered 1:1 mixtures of diastereomers **2f** and **2g** in good yields. L-Proline, which can generate a cyclic iminium intermediate, did not affect diastereotopic selection (19% yield, 51:49 d.r.). Unfortunately, the structure of *α*-amino acid had no definite effect on diastereoselectivity of the Ugi products derived from **1**. Thus, we decided to use 3-formyl DMAP, a related aldehyde component with the intent of improving diastereoselectivity.

### 2.2. Diastereoselective Ugi Reaction of 4-(Dimethylamino)-3-pyridinecarboxaldehyde *(**3**)*

Next, we used 4-(dimethylamino)-3-pyridinecarboxaldehyde (**3**) in the diastereoselective Ugi reaction. As shown in Table 3, the Ugi reaction of **3** with L-valine and *tert*-butyl isocyanide in MeOH at 20 °C afforded the Ugi product **4a** in 37% isolated yield with an 84:16 d.r. The low yield of **4a** is assumed to be due to the presence of an adjacent bulky dimethyl amino group, which encumbers the formation of the imine or iminium species derived from **3** and L-valine. However, because of the reaction of DMAP-based aldehyde **3** and L-valine, large dimethyl amino group might fix imine or iminium configuration (*E* or Z isomer) to avoid steric repulsion against dimethyl amino group. Accordingly, a high d.r. could be observed when **3** was used instead **1**. To enhance the formation of the imine or iminium species, we next carried out the reactions under various reaction temperatures. The reaction at 40 °C proceeded to 79% conversion and afforded **4a** in 53% yield with a 90:10 d.r. (entry 2). Higher conversion (>90%) was achieved at 50 and 60 °C delivering **4a** in 57% and 48% isolated yields with 89:11 and 85:15 d.r., respectively. According to these results, the reaction at 50 °C is preferred considering the yield and diastereoselectivity of the product. Although most of the aldehyde was consumed (92% conversion; entry 3), the isolated yield of **4a** remained moderate. Owing to this, we considered the determination of appropriate substrate ratio of the reaction components (aldehyde, *α*-amino acid, and *tert*-butyl isocyanide) to improve the efficiency of the reaction.

**Table 3:** Ugi reaction of **3** at various reaction temperatures. 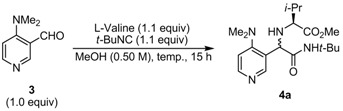

Entry	Temperature (°C)	Conversion (%) *^a^*	Yield (%) *^b^*	D.r. *^a^*
1	20	50	37	84:16
2	40	79	53	90:10
3	50	92	57	89:11
4	60	97	48	85:15

^a^ Conversion and diastereomeric ratio were determined by ^1^H-NMR analysis of unpurified products; ^b^ Yield of the mixture of diastereomers after column chromatography.

To address the aforementioned issue, the substrate ratio was screened under the previously mentioned conditions (Table 4). Although all reactions proceeded in >90% conversion with excess L-valine (1.3 and 1.5 equiv.; entries 2 and 3, respectively) and excess *tert*-butyl isocyanide (1.3 and 1.5 equiv.; entries 4 and 5), the isolated yield was almost same as that of the control reaction (entry 1). The reason for the relatively low isolated yield of the Ugi products with respect to consumption of the starting aldehyde is unclear.

**Table 4:** Substrate ratios for diastereoselective Ugi reaction of **3**. 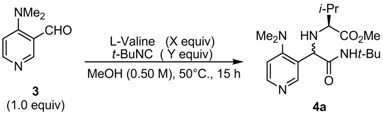

Entry	X	Y	Conversion (%) *^a^*	Yield (%) *^b^*	D.r. *^a^*
1	1.1	1.1	91	54	91:9
2	1.3	1.1	94	55	89:11
3	1.5	1.1	97	55	89:11
4	1.1	1.3	96	59	89:11
5	1.1	1.5	95	57	85:15

^a^ Conversion and diastereomeric ratio were determined by ^1^H-NMR analysis of unpurified products; ^b^ Yield of the mixture of diastereomers after column chromatography.

**Figure 1:**
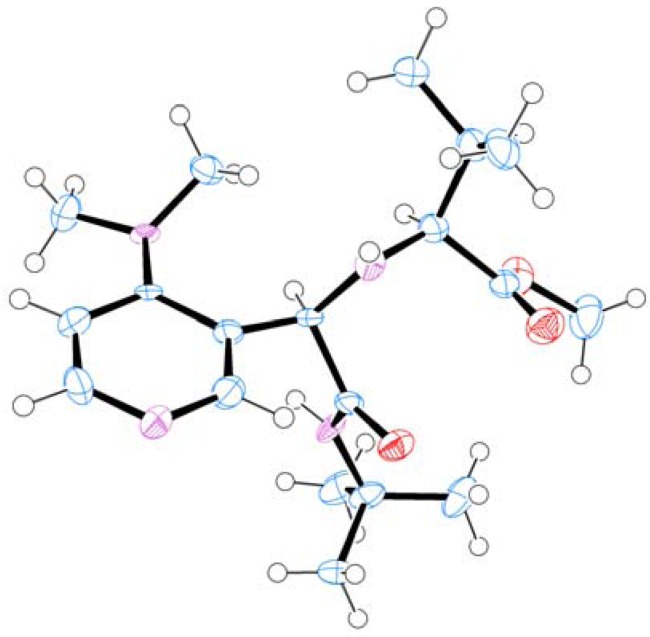
X-ray structure of major diastereomer of **4a**; disordered atoms omitted for clarity.

Considering a relatively effective diastereoselective Ugi reaction of DMAP-based aldehyde **3**, various α-amino acids were investigated under the optimized reaction conditions (Table 5). Reactions with α-amino acids bearing alkyl side chains led to the corresponding products in moderate yield with good to high diastereoselectivity (45%–63%; 59:41–93:7 d.r; entries 1 and 4–8). Ciufolini reported that Ugi reactions of aromatic aldehydes, α-amino acids and *t*-BuNC in MeOH with TiCl_4_ as a catalystshowed improved isolated yields of Ugi product [22]. TiCl_4_ was thus added to the reaction mixture, however the result was almost identical to that obtained under the uncatalyzed reaction (entry 2 *vs.* 1). Furthermore, using MgSO_4_ as a dehydrating agent resulted in slightly decreasing in isolated yield and diastereoselectivity (entry 3 *vs.* 1). Side chains having a heteroatom also showed the same results as those of their alkyl counterparts (entries 9–16). It is surprising that the d.r. of the Ugi products was not dramatically changed by modifying the structure of α-amino acid. Furthermore, the reaction proceeding through the cyclic iminium intermediate derived from L-proline and aldehyde **3** did not improve both yield and diastereoselectivity (51% yield; 74:26 d.r.; entry 17). The stereochemistry of major diastereomer **4a** was determined by X-ray structure analysis, showing *R* configuration at newly formed stereogenic center (Figure 1). The absolute configuration of the major product was different from related reaction reported by Ciufolini [22].

**Table 5:** Ugi reaction of **3** with various *α*-amino acids. 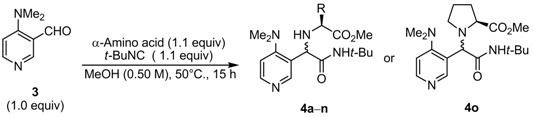

Entry	α-Amino acid	Product	Yield *^a^*	D.r. *^b^*
1	L-Valine	**4a**	55	92:8
2 *^c^*	L-Valine	**4a**	57	88:12
3 *^d^*	L-Valine	**4a**	46	85:15
4	L-Leucine	**4b**	63	86:14
5	L- *t*-Leucine	**4c**	52	89:11
6	L-Isoleucine	**4d**	55	93:7
7	L-Phenylalanine	**4e**	60	88:12
8	L-Phenylglycine	**4f**	45	59:41
9	L-Serine	**4g**	43	77:23
10	L-Methionine	**4h**	58	83:17
11	*O*-*t*-Butyl L-threonine	**4i**	63	82:18
12	γ-Methyl L-glutamate	**4j**	47	83:17
13	4-Benzyl L-aspartate	**4k**	52	79:21
14	*O*-Benzyl L-serine	**4l**	54	75:25
15	L-Histidine	**4m**	44	77:23
16 *^e^*	*N*-Benzyl L-valine	**4n**	18	92:8
17	L-Proline	**4o**	51	74:26

^a^ Yield of the mixture of diastereomer after column chromatography; ^b^ Diastereomeric ratio was determined by ^1^H-NMR analysis of unpurified products; ^c^ 20 mol % of TiCl_4_ was added; ^d^ The suspension of the aldehyde, *α*-amino acid, and MgSO_4_ were stirred for 3 h at room temperature before the addition of *t*-BuNC; ^e^ Reaction time was 48 h.
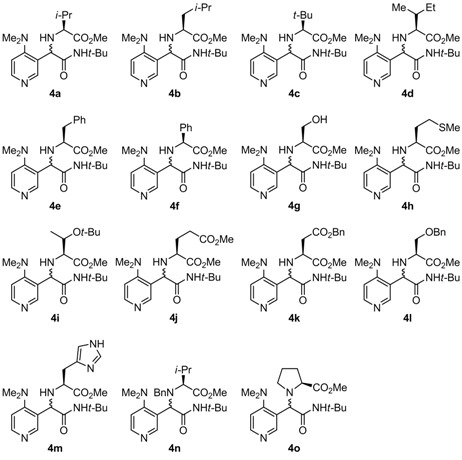

### 2.3. Kinetic Resolution of a Racemic Alcohol by Chiral DMAP Derivatives

**Scheme 1:**
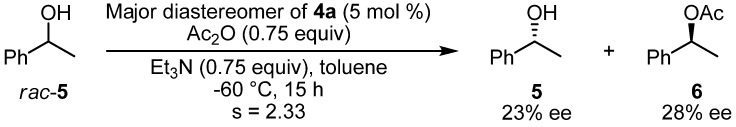
Kinetic resolution of *rac*-**5** using major diastereomer of **4a**.

Next, we explored the possibility of using Ugi products as chiral nucleophilic catalysts. The kinetic resolution of racemic alcohol was selected as a model study. A major diastereomer of Ugi product **4a**, which can be easily obtained by column chromatography, was utilized as the catalyst in toluene at −60 °C ( Scheme 1). The selectivity factor [34], which was estimated by *ee* of acetylated product **6** and unreacted alcohol **5** [35], was indicated to be 2.33. It was noted that the Ugi product **4a** was capable of catalyzing the acylation reaction, although the selectivity was not satisfied at the moment. Further study to develop more efficient catalysts is now under way.

## 3. Experimental

### 3.1. General

All melting points were determined using a Yanaco micro melting point apparatus MP-S3 and are uncorrected. Solvents were generally distilled and dried by standard literature procedures prior to use. The IR spectra were recorded on a JASCO FT/IR-4100 spectrometer. NMR spectra were recorded on a Varian VNMRS-400 spectrometer at SC-NMR Laboratory (Okayama University), operating at 400 MHz for ^1^H-NMR and 100 MHz for ^13^C-NMR. Chemical shifts in CDCl_3_ were reported in the δ scale relative to CHCl_3_ (7.26 ppm) as an internal reference for ^1^H-NMR. For ^13^C-NMR, chemical shifts were reported in the δ scale relative to CHCl_3_ (77.0 ppm) as an internal reference. Column chromatography was performed with silica gel 60 N (spherical, neutral, 40**–**50 μm) purchased from Kanto Chemical. Optical rotations were measured on a Horiba Model SEPA-300 High-sensitive polarimeter. FAB mass spectra (for HRMS) were measured on a JEOL JMS-700 MStation at the Mass Spectrometry Facility (Okayama University). The enantiomeric excess (*ee*) was determined by HPLC analysis. HPLC was performed on Shimadzu HPLC systems consisting of the following: pump, LC-10AD; detector, SPD-10A, 254 nm; column, Daicel Chiracel OD-H; mobile phase, hexane/2-propanol.

### 3.2. General Procedure for the Ugi Reaction of 4-(Dimethylamino)-2-pyridinecarboxaldehyde *(**1**)*

To a suspension of L-valine (23.1 mg, 0.20 mmol) and aldehyde **1** (36.6 mg, 0.24 mmol) in dry methanol (0.4 mL) in a screw-cap test tube, *tert*-butyl isocyanide (17.2 mg, 0.21 mmol) was added and the reaction mixture was stirred for 15 h at room temperature. The solvent was evaporated *in vacuo* and the resulting residue was purified by silica gel column chromatography (EtOAc/Et_3_N = 97:3, v/v) to give (1*R*) *N*-(1-(*N*-*tert*-butylcarbamoyl)-1-(4-(dimethylamino)pyridyn-2-yl)methyl)-L-valine methyl ester (**2a**) as a brown gummy oil (70.5 mg, 98% yield, d.r. 63:37). For mixture of two diastereomers: IR (neat) υ = 3327, 2964, 1738, 1681, 1603 cm*^–^*^1^; ^1^H-NMR (CDCl_3_, major diastereomer) δ 1.03 (d, *J* = 6.0 Hz, 6H), 1.32 (s, 9H), 2.03 (sext, *J* = 6.8 Hz, 1H), 2.03 (br, 1H), 2.97 (s, 6H), 3.02*–*3.07 (m, 1H), 3.60 (s, 3H), 3.90 (s, 1H), 6.39 (dd, *J* = 2.6, 6.0 Hz, 1H), 6.69 (d, *J* = 2.6 Hz, 1H), 7.60 (br, 1H), 8.14 (d, *J* = 6.0 Hz, 1H); ^13^C-NMR (CDCl_3_, major diastereomer) δ 18.4, 19.9, 28.6, 31.5, 39.1, 50.5, 51.5, 65.8, 66.8, 105.1, 105.8, 148.6, 154.6, 156.8, 171.0, 174.9; HRMS-FAB (*m/z*): [M+H]^+^ calcd. for C_19_H_33_N_4_O_3_ 365.2553, found 365.2535.

*(1SR) N-(1-(N-tert-Butylcarbamoyl)-1-(4-(dimethylamino)pyridyn-2-yl)methyl)-L-t-leucine methyl ester* (**2b**): A pale yellow oil (53.6 mg, 70% yield, d.r. 62:38); For mixture of two diastereomers: IR (neat) υ = 3337, 2965, 1732, 1674, 1604 cm^–1^; ^1^H-NMR (CDCl_3_, major diastereomer) δ 1.04 (s, 9H), 1.32 (s, 9H), 2.57 (br, 1H), 2.95 (br, 1H), 2.97 (s, 6H), 3.58 (s, 3H), 3.81 (s, 1H), 6.38 (dd, *J* = 2.4, 6.0 Hz, 1H), 6.66 (d, *J* = 2.4 Hz, 1H), 7.57 (br, 1H), 8.15 (d, *J* = 6.0 Hz, 1H); ^13^C-NMR (CDCl_3_, major diastereomer) δ 26.9, 28.6, 34.2, 39.1, 50.5, 51.1, 67.4, 70.1, 105.1, 105.8, 148.6, 154.6, 156.7, 170.8, 174.5; HRMS-FAB (*m/z*): [M+H]^+^ calcd. for C_20_H_35_N_4_O_3_ 379.2709, found 379.2739.

*(1SR) N-(1-(N-tert-Butylcarbamoyl)-1-(4-(dimethylamino)pyridyn-2-yl)methyl)-L-isoleucine methyl ester* (**2c**): A pale yellow oil (40.5 mg, 54% yield, d.r. 60:40); For mixture of two diastereomers: IR (neat) υ = 2967, 1733, 1673, 1604 cm^–1^; ^1^H-NMR (CDCl_3_, major diastereomer) δ 0.91 (t, *J* = 7.4 Hz, 3H), 0.97 (d, *J* = 6.8 Hz, 3H), 1.31 (s, 9H), 1.55–1.67 (m, 1H), 1.74–1.84 (m, 1H), 2.35 (br, 1H), 2.97 (s, 6H), 3.02–3.06 (m, 1H), 3.15 (d, *J* = 6.0 Hz, 1H), 3.59 (s, 3H), 3.90 (s, 1H), 6.38 (dd, *J* = 2.4, 6.0 Hz, 1H), 6.68 (d, *J* = 2.4 Hz, 1H), 7.60 (br, 1H), 8.14 (d, *J* = 6.0 Hz, 1H); ^13^C-NMR (CDCl_3_, major diastereomer) δ 11.4, 16.2, 25.2, 28.6, 38.2, 39.1, 50.5, 51.5, 65.5, 67.2, 105.1, 105.8, 148.5, 154.6, 156.8, 171.0, 174.8; HRMS-FAB (*m/z*): [M+H]^+^ calcd. for C_20_H_35_N_4_O_3_ 379.2709, found 379.2703.

*(1SR) N-(1-(N-tert-Butylcarbamoyl)-1-(4-(dimethylamino)pyridyn-2-yl)methyl)-L-phenylalanine methylester* (**2d**): A pale yellow oil (45.6 mg, 57% yield, d.r. 55:45); For mixture of two diastereomers: IR (neat) υ = 2964, 1739, 1676, 1604 cm^–1^; ^1^H-NMR (CDCl_3_, major diastereomer) δ 1.09 (s, 9H), 2.81–2.92 (m, 1H), 2.96 (s, 6H), 3.01–3.08 (m, 2H), 3.48–3.54 (m, 1H), 3.57 (s, 3H), 3.95 (s, 1H), 6.38 (dd, *J* = 2.4, 5.6 Hz, 1H), 6.67 (d, *J* = 2.4 Hz, 1H), 7.12 (br, 1H), 7.14–7.31 (m, 5H), 8.14 (d, *J* = 5.6 Hz, 1H); ^13^C-NMR (CDCl_3_, major diastereomer) δ 28.3, 39.1, 39.8, 50.2, 51.7, 62.6, 66.5, 105.0, 105.7, 126.6, 128.5, 129.3, 137.9, 148.5, 154.6, 156.7, 170.6, 174.5; HRMS-FAB (*m/z*): [M+H]^+^ calcd. for C_23_H_33_N_4_O_3_ 413.2553, found 413.2551.

*(1SR) N-(1-(N-tert-Butylcarbamoyl)-1-(4-(dimethylamino)pyridyn-2-yl)methyl)-L-phenylglycine methyl* ester (**2e**): A pale yellow oil (47.6 mg, 59% yield, d.r. 55:45); For mixture of two diastereomers: IR (neat) υ = 2916, 1739, 1673, 1604 cm^–1^; ^1^H-NMR (CDCl_3_, major diastereomer) δ 1.22 (s, 9H), 2.04 (s, 1H), 3.02 (s, 6H), 3.65 (s, 3H), 4.10 (br, 1H), 4.36 (s, 1H), 6.42 (dd, *J* = 2.8, 6.0 Hz, 1H), 6.74 (d, *J* = 2.8 Hz, 1H), 7.25–7.38 (m, 5H), 7.46 (br, 1H), 8.15 (d, *J* = 6.0 Hz, 1H); ^13^C-NMR (CDCl_3_, major diastereomer) δ 28.5, 39.2, 50.7, 52.3, 64.3, 65.5, 105.1, 105.7, 127.8, 128.2, 128.8, 129.8, 137.9, 155.1, 157.0, 170.0, 172.8; HRMS-FAB (*m/z*): [M+H]^+^ calcd. for C_22_H_31_N_4_O_3_ 399.2396, found 399.2409.

*(1SR) N-(1-(N-tert-Butylcarbamoyl)-1-(4-(dimethylamino)pyridyn-2-yl)methyl)-L-threonine methyl ester* (**2f**): A pale yellow oil (61.7 mg, 77% yield, d.r. 50:50); For mixture of two diastereomers: IR (neat) υ = 3329, 2972, 1737, 1673, 1604 cm^–1^; ^1^H-NMR (CDCl_3_, major diastereomer) δ 1.17 (d, *J* = 6.4 Hz, 3H), 1.30 (s, 9H), 1.98 (br, 1H), 3.01 (s, 6H), 3.14 (d, *J* = 6.4 Hz, 1H), 3.65 (s, 3H), 3.95 (quin, *J* = 6.4 Hz, 1H), 4.19 (s, 1H), 4.54 (br, 1H), 6.41 (dd, *J* = 2.4, 6.0 Hz, 1H), 6.68 (d, *J* = 2.4 Hz, 1H), 7.48 (br, 1H), 8.14 (d, *J* = 6.0 Hz, 1H); ^13^C-NMR (CDCl_3_, major diastereomer) δ 19.7, 22.7, 28.5, 39.2, 50.9, 52.0, 66.3, 68.0, 105.1, 105.8, 147.3, 155.2, 156.2, 169.8, 173.5; HRMS-FAB (*m/z*): [M+H]^+^ calcd. for C_18_H_31_N_4_O_4_ 367.2345, found 367.2319.

*(1SR) N-(1-(N-tert-Butylcarbamoyl)-1-(4-(dimethylamino)pyridyn-2-yl)methyl)-L-serine methyl ester* (**2g**): A pale yellow oil (60.3 mg, 85% yield, d.r. 53:47); For mixture of two diastereomers: IR (neat) υ = 3329, 2969, 1739, 1671, 1604 cm^–1^; ^1^H-NMR (CDCl_3_, major diastereomer) δ 1.30 (s, 9H), 2.99 (s, 6H), 3.43 (br, 1H), 3.47–3.50 (m, 1H), 3.66 (s, 3H), 3.75–3.78 (m, 1H), 3.82 (d, *J* = 4.2 Hz, 1H), 4.21 (s, 1H), 4.38 (br, 1H), 6.39 (dd, *J* = 2.8, 6.0 Hz, 1H), 6.70 (d, *J* = 2.8 Hz, 1H), 7.52 (br, 1H), 8.10 (d, *J* = 6.0 Hz, 1H); ^13^C-NMR (CDCl_3_, major diastereomer) δ 28.4, 39.1, 50.8, 52.1, 61.8, 62.3, 65.1, 104.9, 105.6, 147.7, 155.2, 156.8, 170.3, 172.8; HRMS-FAB (*m/z*): [M+H]^+^ calcd. for C_17_H_29_N_4_O_4_ 353.2189, found 353.2200.

#### Procedure for the Synthesis of **2h**

To the mixture of L-proline (33.9 mg, 0.29 mmol) and aldehyde **1** (39.8 mg, 0.27 mmol) in dry methanol (0.4 mL) in a screw-cap test tube, *tert*-butyl isocyanide (30.4 μL, 0.27 mmol) was added and the reaction mixture was stirred for 24 h at room temperature. The solvent was evaporated *in vacuo*. To a solution of the crude product in MeOH (4 mL) was added NaBH_4_ (31.6 mg, 0.84 mmol) at 0 °C owing to reduction of unreacted aldehyde **1**. The reaction mixture was stirred at the same temperature for one hour before being quenched with saturated aq. NH_4_Cl (4 mL). The resulting solution was warmed to room temperature and extracted with Et_2_O (3 × 4 mL). The combined organic phase was washed with brine (15 mL), dried over MgSO_4_, filtered, and concentrated *in vacuo* to give (1*SR*) *N*-(1-(*N*-*tert*-butylcarbamoyl)-1-(4-(dimethylamino)pyridyn-2-yl)methyl)-L-proline methyl ester (**2h**) as a pale yellow oil (18.0 mg, 19% yield, d.r. 51:49). For mixture of two diastereomers: IR (neat) υ = 2967, 1737, 1671, 1603 cm^–1^; ^1^H-NMR (CDCl_3_) δ 1.31 (s, 9H), 1.37 (s, 9H), 1.78–1.89 (m, 6H), 2.07–2.17 (m, 2H), 2.68–2.81 (m, 2H), 2.98 (s, 6H), 2.99 (s, 6H), 3.09–3.15 (m, 2H), 3.57 (s, 3H), 3.63 (s, 3H), 3.65–3.69 (m, 2H), 4.22 (s, 2H), 6.38 (dd, *J* = 2.8, 6.0 Hz, 1H), 6.39 (dd, *J* = 2.8, 6.0 Hz, 1H), 6.51 (d, *J* = 2.8 Hz, 1H), 6.54 (d, *J* = 2.8 Hz, 1H), 7.38 (br, 1H), 7.93 (br, 1H), 8.15 (d, *J* = 6.0 Hz, 1H), 8.17 (d, *J* = 6.0 Hz, 1H); ^13^C-NMR (CDCl_3_) δ 23.6, 23.7, 28.5, 28.6, 29.9, 30.4, 39.1, 39.1, 50.6, 50.7, 50.9, 51.4, 51.5, 52.7, 61.6, 62.9, 74.2, 74.8, 105.6, 105.7, 106.6, 107.1, 149.0, 149.1, 154.7, 154.8, 156.5, 157.1, 170.0, 170.2, 175.4, 175.9; HRMS-FAB (*m/z*): [M+H]^+^ calcd. for C_19_H_31_N_4_O_3_ 363.2396, found 363.2391.

### 3.3. General Procedure for the Ugi Reaction of 4-(Dimethylamino)-3-pyridinecarboxaldehyde *(**3**)*

To the suspension of aldehyde **3** (75.1 mg, 0.50 mmol) and L-valine (64.4 mg, 0.55 mmol) in dry methanol (1.0 mL) in a screw-cap test tube, *tert*-butyl isocyanide (62.0 μL, 0.55 mmol) was added and the reaction mixture was stirred for 15 hours at 50 °C. The solvent was evaporated *in vacuo* and the resulting residue was purified by silica gel column chromatography on SiO_2_ (EtOAc/toluene = 4:1, v/v) to give (1*R*) *N*-(1-(*N*-*tert*-butylcarbamoyl)-1-(4-(dimethylamino)pyridyn-3-yl)methyl)-L-valine methyl ester (**4a**) as a colorless solid (99.7 mg, 55% yield, d.r. 92:8, Table 5, entry 1). For the major diastereomer: m.p. 119 °C; IR (KBr) υ = 3200, 2963, 1737, 1673 cm*^–^*^1^; ^1^H-NMR (CDCl_3_) δ 0.85 (d, *J* = 6.7 Hz, 3H), 0.90 (d, *J* = 6.7 Hz, 3H), 1.34 (s, 9H), 1.92 (sext, *J* = 6.7 Hz, 1H), 2.40 (br, 1H), 2.79 (br, 1H), 2.85 (s, 6H), 3.70 (s, 3H), 4.58 (s, 1H), 6.88 (d, *J* = 5.6 Hz, 1H), 7.26 (br, 1H), 8.36 (d, *J* = 5.6 Hz, 1H), 8.48 (s, 1H); ^13^C-NMR (CDCl_3_) δ 18.4, 19.1, 28.6, 31.3, 44.3, 50.8, 51.5, 58.8, 64.8, 113.6, 127.9, 149.8, 150.3, 159.4, 170.9, 174.6; HRMS-FAB (*m/z*): [M+H]^+^ calcd. for C_19_H_33_N_4_O_3_ 365.2553, found 365.2554; [α]_D_^25^*–*137 (c 0.715, MeOH).

*(1R) N-(1-(N-tert-Butylcarbamoyl)-1-(4-(dimethylamino)pyridyn-3-yl)methyl)-L-leucine methyl ester* (**4b**): A colorless solid (118.6 mg, 63% yield, d.r. 86:14); For the major diastereomer: m.p. 139–141 °C; IR (KBr) υ = 3372, 3210, 2952, 1734, 1666 cm^–1^; ^1^H-NMR (CDCl_3_) δ 0.72 (d, *J* = 6.6 Hz, 3H), 0.85 (d, *J* = 6.6 Hz, 3H), 1.35 (s, 9H), 1.42–1.45 (m, 2H), 1.61 (sext, *J* = 6.6 Hz, 1H), 2.13 (br, 1H, 2.84 (s, 6H), 3.03–3.07 (m, 1H), 3.68 (s, 3H), 4.58 (s, 1H), 6.87 (d, *J* = 5.5 Hz, 1H), 7.37 (br, 1H), 8.35 (d, *J* = 5.5 Hz, 1H), 8.42 (s, 1H); ^13^C-NMR (CDCl_3_) δ 21.7, 22.9, 24.7, 28.6, 42.3, 44.3, 50.9, 51.8, 57.8, 59.0, 113.6, 128.3, 149.9, 150.1, 159.4, 171.0, 175.3; HRMS-FAB (*m/z*): [M+H]^+^ calcd. for C_20_H_35_N_4_O_3_ 379.2709, found 379.2702; [α]_D_^25^ –148 (c 0.205, MeOH).

*(1R) N-(1-(N-tert-Butylcarbamoyl)-1-(4-(dimethylamino)pyridyn-3-yl)methyl)-L-t-leucine methyl ester* (**4c**): A colorless solid (39.6 mg, 52% yield, d.r. 89:11); For the major diastereomer: m.p. 165–166 °C; IR (KBr) υ = 3353, 3214, 2965, 1737, 1677 cm^–1^; ^1^H-NMR (CDCl_3_) δ 0.91 (s, 9H), 1.32 (s, 9H), 2.57–2.66 (m, 2H), 2.84 (s, 6H), 3.68 (s, 3H), 4.49 (s, 1H), 6.89 (d, *J* = 5.5 Hz, 1H), 7.07 (br, 1H), 8.37 (d, *J* = 5.5 Hz, 1H), 8.50 (s, 1H); ^13^C-NMR (CDCl_3_) δ 26.7, 28.7, 34.0, 44.3, 50.8, 51.2, 58.8, 67.7, 113.7, 127.5, 149.9, 150.5, 159.6, 170.8, 174.5; HRMS-FAB (*m/z*): [M+H]^+^ calcd. for C_20_H_35_N_4_O_3_ 379.2709, found 379.2733; [α]_D_^25^ –146 (c 0.270, MeOH).

*(1R) N-(1-(N-tert-butylcarbamoyl)-1-(4-(dimethylamino)pyridyn-3-yl)methyl)-L-isoleucine methyl ester* (**4d**): A colorless solid (104.3 mg, 55% yield, d.r. 93:7); For the major diastereomer: m.p. 97–100 °C; IR (KBr) υ = 3333, 3204, 2967, 1739, 1676 cm^–1^; ^1^H-NMR (CDCl_3_) δ 0.79 (d, *J* = 7.7 Hz, 3H), 0.80 (t, *J* = 7.7 Hz, 3H), 1.06–1.17 (m, 1H), 1.32 (s, 9H), 1.43–1.49 (m, 1H), 1.63–1.69 (m, 1H), 2.37 (br, 1H), 2.83 (s, 6H), 2.86 (d, *J* = 6.0 Hz, 1H), 3.68 (s, 3H), 4.55 (s, 1H), 6.87 (d, *J* = 5.6 Hz, 1H), 7.25 (br, 1H), 8.34 (d, *J* = 5.6 Hz, 1H), 8.45 (s, 1H); ^13^C-NMR (CDCl_3_) δ 11.2, 15.5, 25.2, 28.6, 37.9, 44.3, 50.8, 51.5, 58.9, 63.8, 113.7, 128.0, 149.8, 150.3, 159.4, 170.9, 174.6; HRMS-FAB (*m/z*): [M+H]^+^ calcd. for C_20_H_35_N_4_O_3_ 379.2709, found 379.2713; [α]_D_^25^ –115 (c 0.325, MeOH).

*(1SR) N-(1-(N-tert-Butylcarbamoyl)-1-(4-(dimethylamino)pyridyn-3-yl)methyl)-L-phenylalanine methyl ester* (**4e**): A colorless solid (123.3 mg, 60% yield, d.r. 88:12); For mixture of two diastereomers: m.p. 128–129 °C; IR (KBr) υ = 3319, 3202, 2962, 1739, 1673 cm^–1^; ^1^H-NMR (CDCl_3_, major diastereomer) δ 1.31 (s, 9H), 2.27 (br, 1H), 2.74 (s, 6H), 2.83 (dd, *J* = 7.9, 13.7 Hz, 1H), 2.97 (dd, *J* = 5.9, 13.7 Hz, 1H), 3.32–3.36 (m, 1H), 3.64 (s, 3H), 4.58 (s, 1H), 6.81 (d, *J* = 5.6 Hz, 1H), 7.02–7.04 (m, 2H), 7.18–7.26 (m, 3H), 7.41 (br, 1H), 8.20 (s, 1H), 8.31 (d, *J* = 5.6 Hz, 1H); ^13^C-NMR (CDCl_3_, major diastereomer) δ 28.6, 38.9, 44.3, 50.7, 51.8, 58.8, 60.7, 113.6, 126.8, 127.9, 128.5, 128.9, 136.6, 149.8, 150.0, 159.2, 170.8, 173.9; HRMS-FAB (*m/z*): [M+H]^+^ calcd. for C_23_H_33_N_4_O_3_ 413.2553, found 413.2532.

*(1SR) N-(1-(N-tert-Butylcarbamoyl)-1-(4-(dimethylamino)pyridyn-3-yl)methyl)-L-phenylglycine methyl ester* (**4f**): A colorless solid (90.0 mg, 45% yield, d.r. 59:41); For mixture of two diastereomers: m.p. 146–149 °C; IR (KBr) υ = 3195, 2995, 1738, 1666 cm^–1^; ^1^H-NMR (CDCl_3_, major diastereomer) δ 1.39 (s, 9H), 1.75 (br, 1H), 2.62 (s, 6H), 3.69 (s, 3H), 4.29 (br, 1H), 4.43 (s, 1H), 6.79 (d, *J* = 5.6 Hz, 1H), 7.30–7.40 (m, 5H), 7.43 (br, 1H), 8.31 (d, *J* = 5.6 Hz, 1H), 8.40 (s, 1H); ^13^C-NMR (CDCl_3_, major diastereomer) δ 28.7, 43.9, 51.0, 52.4, 57.9, 64.0, 113.6, 127.8, 128.5, 128.7, 128.9, 137.2, 149.7, 149.8, 158.9, 170.6, 172.4; HRMS-FAB (*m/z*): [M+H]^+^ calcd. for C_22_H_31_N_4_O_3_ 399.2396, found 399.2382.

*(1SR) N-(1-(N-tert-Butylcarbamoyl)-1-(4-(dimethylamino)pyridyn-3-yl)methyl)-L-serine methyl ester* (**4g**): A yellowish solid (76.2 mg, 43% yield, d.r. 77:23); For mixture of two diastereomers: m.p. 121 °C; IR (KBr) υ = 3451, 3202, 2959, 1722, 1666 cm^–1^; ^1^H-NMR (CDCl_3_, major diastereomer) δ 1.35 (s, 9H), 2.86 (br, 1H), 2.86 (s, 6H), 3.29 (dd, *J* = 4.4, 5.3 Hz, 1H), 3.70–3.72 (m, 1H), 3.74 (s, 3H), 3.79–3.85 (m, 1H), 4.71 (s, 1H), 6.92 (d, *J* = 5.6 Hz, 1H), 7.28 (br, 1H), 8.36 (d, *J* = 5.6 Hz, 1H), 8.43 (s, 1H); ^13^C-NMR (CDCl_3_, major diastereomer) δ 28.6, 44.5, 51.1, 52.2, 58.5, 61.0, 62.5, 114.0, 128.5, 149.7, 149.8, 159.3, 170.7, 172.7; HRMS-FAB (*m/z*): [M+H]^+^ calcd. for C_17_H_29_N_4_O_4_ 353.2189, found 353.2175.

*(1R) N-(1-(N-tert-Butylcarbamoyl)-1-(4-(dimethylamino)pyridyn-3-yl)methyl)-L-methionine methyl ester* (**4h**): A colorless solid (115.0 mg, 58% yield, d.r. 83:17). For the major diastereomer: m.p. 98–100 °C; IR (KBr) υ = 3203, 2971, 1735, 1671 cm^–1^; ^1^H-NMR (CDCl_3_) δ 1.26 (s, 9H), 1.70–1.79 (m, 1H), 1.83–1.91 (m, 1H), 1.95 (s, 3H), 2.34–2.49 (m, 2H), 2.35 (br, 1H), 2.77 (s, 6H), 3.14 (br, 1H), 3.63 (s, 3H), 4.57 (s, 1H), 6.82 (d, *J* = 5.5 Hz, 1H), 7.18 (br, 1H), 8.28 (d, *J* = 5.5 Hz, 1H), 8.39 (s, 1H); ^13^C-NMR (CDCl_3_) δ 15.2, 28.5, 30.3, 32.2, 44.2, 50.7, 51.8, 58.0, 58.4, 113.6, 128.0, 149.8, 150.0, 159.1, 170.6, 174.2; HRMS-FAB (*m/z*): [M+H]^+^ calcd. for C_19_H_33_N_4_O_3_S 397.2273, found 397.2245; [α]_D_^25^ −156 (c 0.420, MeOH).

*(1SR) N-(1-(N-tert-Butylcarbamoyl)-1-(4-(dimethylamino)pyridyn-3-yl)methyl) O-t-butyl L-threonine methyl ester* (**4i**): A colorless solid (132.6 mg, 63% yield, d.r. 82:18); For mixture of two diastereomers: m.p. 136 °C; IR (KBr) υ = 3387, 3249, 2977, 1732, 1675 cm^–1^; ^1^H-NMR (CDCl_3_, major diastereomer) δ 1.05 (s, 9H), 1.08 (d, *J* = 6.2 Hz, 3H), 1.35 (s, 9H), 2.20 (br, 1H), 2.54 (br, 1H), 2.84 (s, 6H), 3.68 (s, 3H), 3.86 (td, *J* = 6.2, 10.7 Hz, 1H), 4.65 (s, 1H), 6.84 (d, *J* = 5.6 Hz, 1H), 7.55 (br, 1H), 8.31 (d, *J* = 5.6 Hz, 1H), 8.40 (s, 1H); ^13^C-NMR (CDCl_3_, major diastereomer) δ 20.5, 28.3, 28.6, 44.4, 50.8, 51.7, 59.1, 65.5, 68.2, 74.0, 113.5, 128.2, 149.7, 149.9, 159.5, 171.3, 173.5; HRMS-FAB (*m/z*): [M+H]^+^ calcd. for C_22_H_39_N_4_O_4_ 423.2971, found 423.2973.

*(1SR) N-(1-(N-tert-Butylcarbamoyl)-1-(4-(dimethylamino)pyridyn-3-yl)methyl) L-glutamic acid dimethyl ester* (**4j**): A colorless solid (94.9 mg, 47% yield, d.r. 83:17); For the major diastereomer: m.p. 89–90 °C; IR (KBr) υ = 3202, 2970, 1740, 1672 cm^–1^; ^1^H-NMR (CDCl_3_) δ 1.34 (s, 9H), 1.82–2.02 (m, 2H), 2.30 (br, 1H), 2.35 (t, *J* = 7.6 Hz, 2H), 2.84 (s, 6H), 3.04 (dd, *J* = 5.8, 7.6 Hz, 1H), 3.63 (s, 3H), 3.70 (s, 3H), 4.64 (s, 1H), 6.88 (d, *J* = 5.6 Hz, 1H), 7.11 (br, 1H), 8.36 (d, *J* = 5.6 Hz, 1H), 8.43 (s, 1H); ^13^C-NMR (CDCl_3_) δ 27.9, 28.7, 30.2, 44.4, 51.0, 51.7, 52.0, 58.2, 58.3, 113.7, 127.9, 149.9, 150.0, 159.5, 170.7, 173.2, 174.2; HRMS-FAB (*m/z*): [M+H]^+^ calcd. for C_20_H_33_N_4_O_5_ 409.2451, found 409.2426; [α]_D_^25^ −156 (c 0.120, MeOH).

*(1SR) -Benzyl N-(1-(N-tert-butylcarbamoyl)-1-(4-(dimethylamino)pyridyn-3-yl)methyl)-L-aspartic acid methyl ester* (**4k**): A colorless solid (122.5 mg, 52% yield, d.r. 79:21); For mixture of two diastereomers: m.p. 105 °C; IR (KBr) υ = 3334, 3202, 2971, 1739, 1668 cm^–1^; ^1^H-NMR (CDCl_3_, major diastereomer) δ 1.34 (s, 9H), 1.98 (br, 1H), 2.75 (dd, *J* = 3.0, 6.1 Hz, 2H), 2.81 (s, 6H), 3.52 (br, 1H), 3.66 (s, 3H), 4.67 (s, 1H), 5.06 (dd, *J* =12.2, 18.4 Hz, 2H), 6.87 (d, *J* = 5.5 Hz, 1H), 7.27–7.36 (m, 5H), 7.40 (br, 1H), 8.36 (d, *J* = 5.5 Hz, 1H), 8.41 (s, 1H); ^13^C-NMR (CDCl_3_, major diastereomer) δ 28.6, 36.9, 44.4, 50.9, 52.2, 56.0, 58.4, 66.6, 113.9, 128.3, 128.3, 128.4, 128.5, 135.4, 150.0, 150.0, 159.2, 170.4, 170.7, 172.8; HRMS-FAB (*m/z*): [M+H]^+^ calcd. for C_25_H_35_N_4_O_5_ 471.2607, found 471.2593.

*(1SR) N-(1-(N-tert-Butylcarbamoyl)-1-(4-(dimethylamino)pyridyn-3-yl)methyl) O-benzyl L-serine methyl ester* (**4l**): A colorless solid (120.2 mg, 54% yield, d.r. 75:25); For mixture of two diastereomers: m.p. 145 °C; IR (KBr) υ = 3203, 2969, 1743, 1667 cm^–1^; ^1^H-NMR (CDCl_3_, major diastereomer) δ 1.36 (s, 9H), 1.74 (br, 1H), 2.80 (s, 6H), 3.33 (br, 1H), 3.52–3.65 (m, 2H), 3.71 (s, 3H), 4.41 (dd, *J* = 12.2, 14.3 Hz, 2H), 4.69 (s, 1H), 6.86 (d, *J* = 5.6 Hz, 1H), 7.17–7.19 (m, 2H), 7.24–7.37 (m, 3H), 7.57 (br, 1H), 8.35 (d, *J* = 5.6 Hz, 1H), 8.41 (s, 1H); ^13^C-NMR (CDCl_3_, major diastereomer) δ 28.7, 44.4, 50.9, 52.1, 59.0, 59.6, 70.1, 73.1, 113.8, 127.6, 127.8, 128.4, 128.5, 137.5, 149.8, 150.0, 159.4, 171.0, 172.4; HRMS-FAB (*m/z*): [M+H]^+^ calcd. for C_24_H_35_N_4_O_4_ 443.2658, found 443.2681.

*(1SR) N-(1-(N-tert-Butylcarbamoyl)-1-(4-(dimethylamino)pyridyn-3-yl)methyl)-L-histidine methyl ester* (**4m**): A colorless solid (88.1 mg, 44% yield, d.r. 77:23); For mixture of two diastereomers: m.p. 110–113 °C; IR (KBr) υ = 3320, 3206, 2960, 1741, 1676 cm^–1^; ^1^H-NMR (CDCl_3_, major diastereomer) δ 1.33 (s, 9H), 2.55 (br, 1H), 2.81 (s, 6H), 2.86–3.06 (m, 2H), 3.42 (dd, *J* = 5.1, 7.6 Hz, 1H), 3.70 (s, 3H), 4.65 (s, 1H), 6.67 (s, 1H), 6.86 (d, *J* = 5.6 Hz, 1H), 7.44 (s, 1H), 7.46 (br, 1H), 8.23 (s, 1H), 8.29 (d, *J* = 5.6 Hz, 1H); ^13^C-NMR (CDCl_3_, major diastereomer) δ 28.6, 28.6 30.1, 44.3, 44.4, 50.9, 52.1, 58.7, 59.7, 113.7, 128.5, 135.1, 149.5, 149.6, 159.4, 171.0, 174.0; HRMS-FAB (*m/z*): [M+H]^+^ calcd. for C_20_H_31_N_6_O_3_ 403.2458, found 403.2460.

*(1SR) N-Benzyl-(1-(N-tert-butylcarbamoyl)-1-(4-(dimethylamino)pyridyn-3-yl)methyl)-L-valine methyl ester* (**4n**): A colorless syrup (15.9 mg, 18% yield, d.r. 92:8); For mixture of two diastereomers: IR (KBr) υ = 3362, 2966, 1732, 1673 cm^–1^; ^1^H-NMR (CDCl_3_, major diastereomer) δ 0.65 (d, *J* = 6.6 Hz, 3H), 0.75 (d, *J* = 6.6 Hz, 3H), 1.37 (s, 9H), 1.89–2.05 (m, *J* = 6.6 Hz, 1H), 2.74 (s, 6H), 2.84 (s, 1H), 3.70 (d, *J* = 14.4 Hz, 1H), 3.79 (s, 3H), 4.30 (d, *J* = 14.4 Hz, 1H), 5.18 (s, 1H), 6.85 (d, *J* = 5.6 Hz, 1H), 6.90 (br, 1H), 7.19–7.28 (m, 5H), 8.28 (d, *J* = 5.6 Hz, 1H), 8.47 (s, 1H); ^13^C-NMR (CDCl_3_, major diastereomer) δ 19.7, 20.4, 28.7, 44.2, 51.1, 51.3, 53.4, 60.8, 68.7, 113.3, 127.1, 128.2, 128.6, 139.4, 149.1, 149.8, 150.3, 152.1, 159.4, 170.3, 174.8; HRMS-FAB (*m/z*): [M+H]^+^ calcd. for C_26_H_39_N_4_O_3_ 455.3022, found 455.3043.

*(1SR) N-(1-(N-tert-Butylcarbamoyl)-1-(4-(dimethylamino)pyridyn-3-yl)methyl)-L-proline methyl ester* (**4o**): A yellowish solid (93.5 mg, 51% yield, d.r. 74:26); For mixture of two diastereomers: m.p. 105–109 °C; IR (KBr) υ = 3276, 2967, 1734, 1663 cm^–1^; ^1^H-NMR (CDCl_3_, major diastereomer) δ 1.38 (s, 9H), 1.69–1.87 (m, 3H), 1.99–2.09 (m, 1H), 2.27–2.33 (m, 1H), 2.76–2.81 (m, 1H), 2.87 (s, 6H), 3.46 (dd, *J* = 3.6, 9.5 Hz, 1H), 3.69 (s, 3H), 4.89 (s, 1H), 6.86 (d, *J* = 5.6 Hz, 1H), 7.50 (br, 1H), 8.32 (d, *J* = 5.6 Hz, 1H), 8.41 (s,1H); ^13^C-NMR (CDCl_3_, major diastereomer) δ 23.7, 28.7, 29.6, 44.6, 50.0, 50.9, 51.7, 63.1, 63.9, 113.6, 125.4, 149.4, 151.3, 160.4, 170.7, 175.1; HRMS-FAB (*m/z*): [M+H]^+^ calcd. for C_19_H_31_N_4_O_3_ 363.2396, found 363.2399.

### 3.4. Kinetic Resolution of a Racemic Alcohol by Chiral DMAP Derivative

To a solution of major diastereomer of **4a** (3.6 mg, 0.01 mmol), 1-phenylethanol (**5**, 25.4 μL, 0.21 mmol) and triethylamine (21.0 μL, 0.15 mmol) in toluene (0.4 mL) at –60 °C, was added acetic anhydride (14.0 μL, 0.15 mmol) and the reaction mixture was stirred at the same temperature for 15 h. The reaction was quenched with methanol and concentrated *in vacuo*. The resulting residue was filtered through a short plug of silica gel (hexane/Et_2_O = 3:1, v/v), affording a 28% *ee* of (*S*) acetate and a 23% *ee* of unreacted (*R*) alcohol at 43% conversion determined by ^1^H-NMR analysis. The *ee* values indicate s = 2.33 at 43% conversion. HPLC (DAICEL CHIRALCEL OD-H, 0.46 cm × 25 cm, hexane/isopropanol = 19:1; 0.3 mL/min; 30 °C): *R*_t_ 14.5 min (minor ester), 15.3 min (major ester), 27.9 min (major alcohol), and 31.8 min (minor alcohol).

### 3.5 X-ray Structure Report for (**S,R**)***-4a***

CCDC 833504 contains the supplementary crystallographic data for this paper. These data can be obtained free of charge via www.ccdc.cam.ac.uk/conts/retrieving.html.

#### 3.5.1. Data Collection

A colorless prism crystal of C_19_H_32_N_4_O_3_ having approximate dimensions of 0.600 × 0.080 × 0.060 mm was mounted on a glass fiber. All measurements were made on a Rigaku Saturn724 diffractometer using multi-layer mirror monochromated Mo-Ka radiation. The data were collected at a temperature of −179 ± 1 °C to a maximum 2q value of 55.0°. A total of 1440 oscillation images were collected. A sweep of data was done using w oscillations from −110.0 to 70.0° in 0.5° steps. The exposure rate was 20.0 [s/°]. The detector swing angle was −19.76°. A second sweep was performed using w oscillations from −110.0 to 70.0° in 0.5° steps. The exposure rate was 20.0 [s/°]. The detector swing angle was −19.76°. Another sweep was performed using w oscillations from −110.0 to 70.0° in 0.5° steps. The exposure rate was 20.0 [s/°]. The detector swing angle was −19.76°. Another sweep was performed using w oscillations from −110.0 to 70.0° in 0.5° steps. The exposure rate was 20.0 [s/°]. The detector swing angle was −19.76°. The crystal-to-detector distance was 44.98 mm. Readout was performed in the 0.141 mm pixel mode (Table 6).

**Table 6:** Crystal data and structure refinement for ydkr.

Empirical formula	C19 H32 N4 O3	
Formula weight	364.49	
Crystal Color, Habit	colorless, needle	
Temperature	93(2) K	
Wavelength	0.71075 Å	
Crystal system	orthorhombic	
Space group	P2_1_2_1_2 (#18)	
Unit cell dimensions	a = 17.791(6) Å	a = 90°
	b = 17.955(6) Å	b = 90°
	c = 6.363(2) Å	g = 90°
Volume	2032.6(11) Å3	
Z	4	
Density (calculated)	1.191 Mg/m^3^	
Absorption coefficient	0.082 mm^−1^	
F(000)	792	
Crystal size	0.60 × 0.08 × 0.06 mm^3^	
Theta range for data collection	3.22 to 27.49°.	
Index ranges	−23 ≤ h ≤ 23, −23 ≤ k ≤ 23, −8 ≤ l ≤ 8	
Reflections collected	31451	
Independent reflections	4665 [R(int) = 0.0603]	
Completeness to theta = 27.49°	99.60%	
Refinement method	Full-matrix least-squares on F2	
Data / restraints / parameters	4665 / 816 / 487	
Goodness-of-fit on F2	0.966	
Final R indices [I > 2sigma(I)]	R1 = 0.0471, wR2 = 0.1109	
R indices (all data)	R1 = 0.0619, wR2 = 0.1202	
Absolute structure parameter	0.1(12)	
Extinction coefficient	0.024(3)	
Largest diff. peak and hole	0.228 and −0.238 e.Å^−3^	

#### 3.5.2. Data Reduction

Of the 31,451 reflections that were collected, 4,665 were unique (Rint= 0.0603); equivalent reflections were merged. Data were collected and processed using CrystalClear (Rigaku) [36].

#### 3.5.3. Structure Solution and Refinement

The structure was solved by direct methods [37] and expanded using Fourier techniques. The non-hydrogen atoms were refined anisotropically. Hydrogen atoms were refined using the riding model. The final cycle of full-matrix least-squares refinement [38] on F [39] was based on 4665 observed reflections and 236 variable parameters and converged (largest parameter shift was 0.00 times its esd) with unweighted and weighted agreement factors of:

R1 = S ||Fo| − |Fc|| / S |Fo| = 0.0471

wR2 = [S(w (Fo^2^ – Fc^2^)^2^)/ S w(Fo^2^)^2^]^1/2^ = 0.1109

The standard deviation of an observation of unit weight [40] was 1.50. Unit weights were used. The maximum and minimum peaks on the final difference Fourier map corresponded to 1.48 and −0.80 e^−^/Å3, respectively. The absolute structure was deduced based on Flack parameter, −2(4), using 1983 Friedel pairs [41].

Neutral atom scattering factors were taken from Cromer and Waber [42]. Anomalous dispersion effects were included in Fcalc [43]; the values for Df' and Df" were those of Creagh and McAuley [44]. The values for the mass attenuation coefficients are those of Creagh and Hubbell [45]. All calculations were performed using the Yadokari-XG 2009 [46] crystallographic software package except for refinement, which was performed using SHELXL-97 [47].

## 4. Conclusions

We have studied the diastereoselective Ugi reactions of DMAP-based aldehydes with α-amino acids and *tert*-butyl isocyanide. The reactions of 4-(dimethylamino)-2-pyridinecarboxaldehyde (**1**) with various α-amino acids as a chiral source proceeded to afford the desired Ugi products **2a**–**h** in moderate to high yield (19%–98%) with low diastereoselectivity ratio (50:50–63:37; d.r.), even though various α-amino acids structures were investigated. On the other hand, the reactions of 4-(dimethylamino)-3-pyridinecarboxaldehyde (**3**) with various α-amino acids delivered the desired Ugi products **4a**–**o** in moderate yield (18%–63%) with high diastereoselectivity (up to 93:7 d.r.). The fact that the combination of α-amino acid and 3-formyl DMAP is critical to achieve high diastereoselectivity is noteworthy. We also demonstrated that the kinetic resolution of racemic alcohol **5** using a major diastereomer of the Ugi product **4a** as a catalyst afforded enantioenriched acetylated product **6** and unreacted alcohol **5** with a selectivity factor of 2.33. The result indicated that the Ugi products are potential chiral nucleophilic catalysts. Further optimization of the catalyst structure and the application of these catalysts to other important asymmetric transformations are currently in progress.
